# Individual participant data meta‐analysis versus aggregate data meta‐analysis: A case study in eczema and food allergy prevention

**DOI:** 10.1111/cea.14085

**Published:** 2022-01-10

**Authors:** Eleanor Van Vogt, Suzie Cro, Victoria R. Cornelius, Hywel C. Williams, Lisa M. Askie, Rachel Phillips, Maeve M. Kelleher, Robert J. Boyle

**Affiliations:** ^1^ Imperial Clinical Trials Unit Imperial College London London UK; ^2^ Centre of Evidence Based Dermatology University of Nottingham Nottingham UK; ^3^ NHMRC Clinical Trials Centre University of Sydney Camperdown New South Wales Australia; ^4^ National Heart and Lung Institute Section of Inflammation and Repair Imperial College London London UK

**Keywords:** atopic dermatitis, food allergy, individual participant data Introduction, meta‐analysis, prevention

## Abstract

**Introduction:**

Meta‐analysis traditionally uses aggregate data from published reports. Individual Participant Data (IPD) meta‐analysis, which obtains and synthesizes participant‐level data, is potentially more informative, but resource‐intensive. The impact on the findings of meta‐analyses using IPD in comparison with aggregate data has rarely been formally evaluated.

**Methods:**

We conducted a secondary analysis of a Cochrane systematic review of skincare interventions for preventing eczema and food allergy in infants to identify the impact of the analytical choice on the review's findings. We used aggregate data meta‐analysis only and contrasted the results against those of the originally published IPD meta‐analysis. All meta‐analysis used random effects inverse variance models. Certainty of evidence was evaluated using GRADE.

**Results:**

The pooled treatment effects for the Cochrane systematic review's co‐primary outcomes of eczema and food allergy were similar in IPD meta‐analysis (eczema RR 1.03, 95% CI 0.81, 1.31; I^2^41%, 7 studies 3075 participants), and aggregate meta‐analysis (eczema RR 1.01 95% CI 0.77, 1.33; I^2^53%, 7 studies, 3089 participants). In aggregate meta‐analysis, the statistical heterogeneity could not be explained but using IPD it was explained by one trial which used a different, bathing intervention. For IPD meta‐analysis, risk of bias was assessed as lower and more adverse event data were available compared with aggregate meta‐analysis. This resulted in higher certainty of evidence, especially for adverse events. IPD meta‐analysis enabled analysis of treatment interactions by age and hereditary eczema risk; and analysis of the effect of treatment adherence using pooled complier‐adjusted‐causal‐effect analysis, none of which was possible in aggregate meta‐analysis.

**Conclusions:**

For this systematic review, IPD did not significantly change primary outcome risk ratios compared with aggregate data meta‐analysis. However, certainty of evidence, safety outcomes, subgroup and adherence analyses were significantly different using IPD. This demonstrates benefits of adopting an IPD approach to meta‐analysis.

## Graphical abstract

Benefits of an IPD approach to meta‐analysis were demonstrated. Risk ratios were similar, but IPD allowed a conclusion that skincare interventions probably do not affect eczema risk, compared with a conclusion that they may not affect eczema risk when using aggregate data meta‐analysis, due to unexplained statistical heterogeneity in the latter. GRADE certainty of evidence ratings, adverse events analyses, subgroup and adherence analyses were either significantly different with IPD or only possible with IPD compared with aggregate data meta‐analysis.

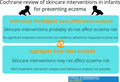

**Key messages**

- We compared IPD and aggregate meta‐analysis using data from a Cochrane review of skincare interventions.
- Primary outcome estimates were similar, but certainty of evidence increased for Individual Participant Data meta‐analysis.
- Safety, subgroup and adherence analysis and heterogeneity investigation were also facilitated with Individual Participant Data.

## 1 INTRODUCTION

Meta‐analysis is fundamental to evidence‐based decision making, combining quantitative outcomes across multiple related studies to summarize all the available evidence on a particular clinical question. Traditional meta‐analysis combines aggregate data (e.g. mean difference, risk ratio, etc.) obtained from individual trial publications or trial authors. Individual Participant Data (IPD) refers to raw, original participant level data. In an IPD meta‐analysis, IPD is collected from all possible eligible trials and included in the synthesis. Data sharing has become more common in recent years meaning easier implementation and increased use of IPD meta‐analysis.^1^, ^2^

Individual Participant Data meta‐analysis is considered ‘the gold standard of systematic reviews’. There are many well‐known potential advantages of IPD meta‐analysis over aggregate meta‐analysis.^2^, ^3^, ^4^ These include the ability to standardize participant inclusion and exclusion criteria and outcome definitions across studies, use of uniform statistical methods, such as consistent adjustment for baseline characteristics, analysis models and methods of handling missing data. Other benefits include the ability to explore how a treatment effect is modified by participant factors, and more in‐depth risk of bias assessments. IPD from unpublished studies can also be included, reducing publication bias. Reviewers can independently check the trial data set for errors and recalculate poorly reported outcomes from published studies. However, it is more resource intensive, requiring large collaborations, trusting relationships and data sharing in addition to more complex data preparation and analysis.^3^, ^5^, ^6^

In this article, we investigated the potential added value of conducting IPD meta‐analysis for a previously conducted IPD Cochrane systematic review which primarily assessed the effects of skincare interventions, such as emollients, for primary prevention of eczema and food allergy in infants.^7^ Secondary objectives of the original systematic review were to identify features of the study population associated with greater treatment benefit or harm including age, hereditary risk and intervention adherence. Using the same search results, we conducted a secondary analysis of the review and meta‐analysis using aggregate methods only and compared the results to those of the previously reported primary IPD meta‐analysis. We sought to identify the impacts of the analytical choice on the review's findings, in order to help guide other researchers planning future meta‐analysis or interpreting meta‐analysis results.

## 2 METHODS

### 2.1 Outcomes

The main outcomes of interest were (i) differences in the quantity of studies included within IPD vs traditional aggregate data meta‐analyses, (ii) differences between the pooled treatment effects sizes from IPD vs. traditional meta‐analysis for the two co‐primary outcomes (described below). Additional outcomes were (i) differences between the pooled treatment effects sizes from IPD and traditional meta‐analysis for secondary outcomes (described below), (ii) pooled effect sizes that were only possible with IPD (iii) differences in risk of bias assessments, and (iv) differences in quality of the evidence. We also summarize the costs and resources involved for the IPD‐analysis.

The co‐primary Cochrane review outcomes were (i) eczema by 1–3 years assessed using the Hanifin and Rajka criteria^8^ or the UK Working Party refinement of them^9^ and (ii) food allergy by 1–3 years assessed using a combination of parental history, skin prick tests and if needed, oral food challenge. If (i) was not available then doctor diagnosis of eczema could be used; if no doctor diagnosis was available, then parent report of eczema was used. Secondary outcomes were: skin infections; stinging or application site reactions to moisturizers; slippage accidents; serious adverse events; clinically‐assessed eczema severity at 1–3 years; parent report of eczema severity at 1–3 years; time to eczema onset; parent report of immediate (<2 hours) reaction to a known food allergen at 1–3 years; and allergic sensitization to foods and inhalants at 1–3 years.

### 2.2 Types of studies, participants and interventions

The types of studies, participants and interventions eligible for inclusion within the review which we re‐analyse here have previously been reported.^10^ To summarize, eligible studies were parallel‐group, or factorial randomized trials using individual or cluster randomization. Participants were infants aged from birth to 12 months, excluding study populations defined by pre‐existing disease or illness. Interventions were skin care interventions that could potentially enhance the skin barrier function, reduce dryness or subclinical inflammation such as moisturizers/emollients, bathing products, advice regarding soap exposure and bathing frequency. It is thought that disruption of the skin barrier in early life can lead to eczema and subsequent food sensitization and allergy. Comparators were no treatment intervention or standard care in the study setting.

### 2.3 Search strategy

We used the same search of MEDLINE, Embase, CENTRAL and trial registers (performed July 23rd 2020) and study selection as the Cochrane review previously reported.^10^ Two review authors independently screened study titles and abstracts for eligibility, with arbitration by a third author where necessary. The full texts of all potentially eligible studies from the search were obtained to confirm eligibility. No language restrictions were imposed.

### 2.4 Data extraction

Aggregate data were extracted from trial publications of the eligible studies and did not include any author correspondence. Data were extracted into excel in duplicate by EVV and SC. Any differences in extraction were discussed and resolved.

### 2.5 Statistical analysis

Aggregate meta‐analyses included eligible trials providing aggregate data for the relevant outcome. For binary outcomes, we calculated a pooled Risk Ratio (RR), for continuous outcomes a pooled standardized mean difference (SMD), and for time to event outcomes a pooled Hazard Ratio (HR). All estimates had associated 95% Confidence Intervals (CI) calculated.

Aggregate meta‐analyses were carried out using inverse variance random effects models. As the majority of trials only reported unadjusted outcomes, we combined unadjusted estimates for the primary aggregate analysis. Aggregate meta‐analysis, including adjusted RR estimates, was conducted for sensitivity analysis where possible. Across meta‐analyses, the I² statistic and Chi^2^ test quantify the degree of statistical heterogeneity of trials judged as clinically homogeneous.^11^ Analysis was carried out in STATA 15 and Revman.

### 2.6 Risk of bias assessment and quality of evidence

Two authors (SC and RJB) re‐assessed risk of bias for outcomes with risk of bias assessments in the IPD meta‐analysis using the aggregate data to mimic a traditional meta‐analysis. Aggregate data risk of bias assessments were compared to original IPD assessments. The Cochrane ‘Risk of bias 2’ tool was used. The GRADE approach was applied to the main outcomes of the Cochrane review by two authors (SC and RJB), using aggregate data information to compare to the IPD assessments. Outcomes were graded as high, moderate, low or very low quality.

### 2.7 Additional methods of previously conducted IPD analysis

The protocol for the original Cochrane IPD review has previously been published.^7^ A formal protocol was not prepared for the aggregate data secondary analysis. In the Cochrane IPD review, all trial authors of eligible studies were contacted and asked to provide IPD. Data were de‐identified, transferred, then cleaned and coded for analysis. Consistency checks against published results were carried out on the data, any errors or extreme values were queried with trial authors where necessary.

For the IPD meta‐analysis, a pre‐specified statistical analysis plan (SAP) was previously followed.^12^ No new IPD meta‐analysis was performed for this article. In brief, the previously conducted IPD meta‐analysis used a two‐stage approach. In stage 1, for binary/continuous/time‐to‐event outcomes, a binomial/linear/binomial with a complementary log‐log link regression model was, respectively, used. All stage 1 models included sex and where relevant (trial not exclusively in a high risk population) family history of atopic disease and resulted in adjusted RR. In the second stage, inverse variance random‐effects models were used to obtain a pooled treatment effect. As the aggregate meta‐analysis predominately used unadjusted treatment estimates, we did not expect aggregate and IPD meta‐analysis results to be identical.

## 3 RESULTS

### 3.1 Number of studies in aggregate and IPD meta‐analysis

**FIGURE 1:**
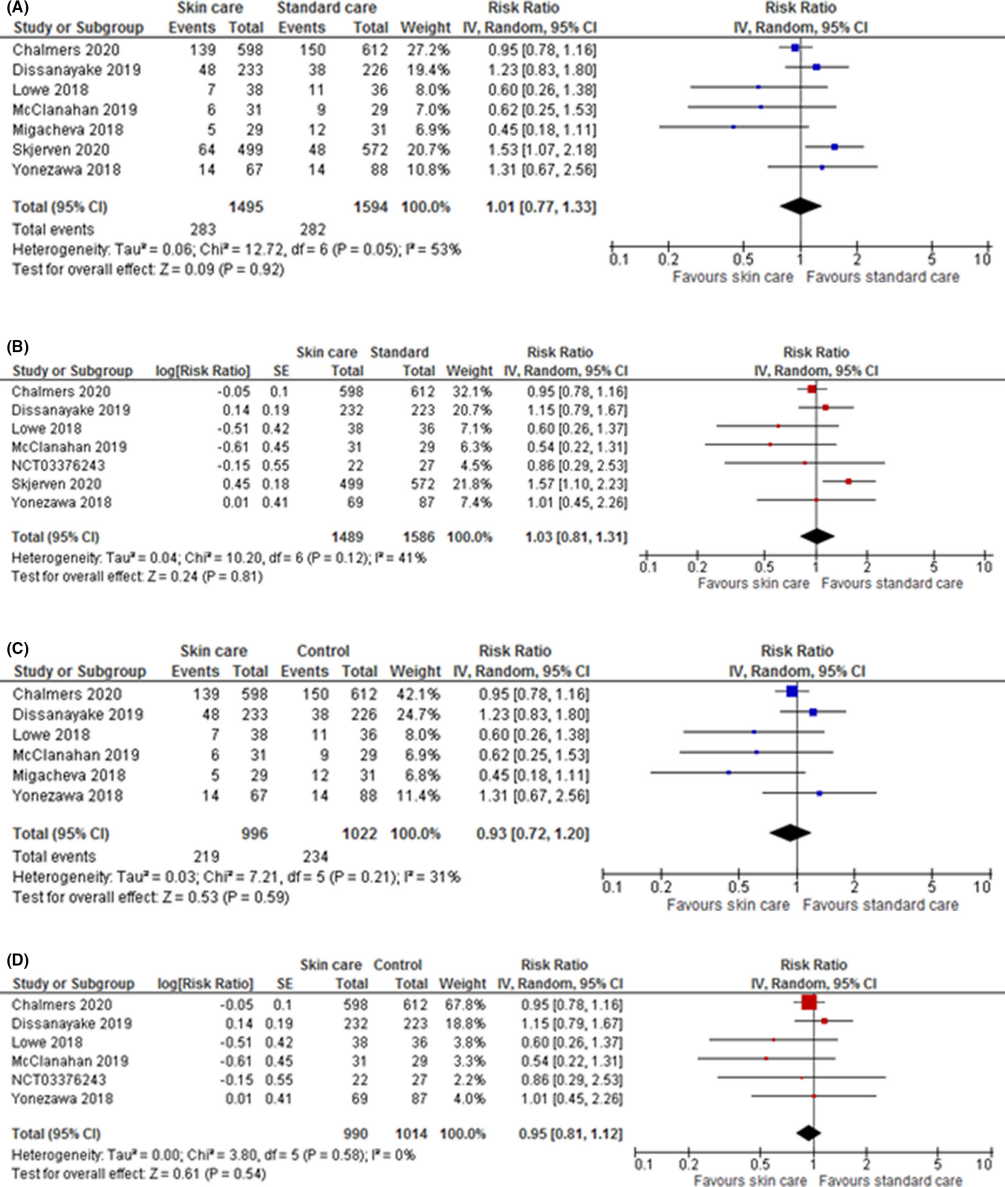
IPD meta‐analysis forest plots versus aggregate meta‐analysis forest plots for skin care intervention versus standard skin care or no skin care intervention for eczema by 1–3 years. (A) Aggregate meta‐analysis for skin care intervention versus standard skin care or no skin care intervention for eczema by 1–3 years. (B) IPD meta‐analysis for skin care intervention versus standard skin care or no skin care intervention for eczema by 1–3 years. (C) Aggregate meta‐analysis excluding Skjerven sensitivity analysis. (D) IPD meta‐analysis excluding Skjerven sensitivity analysis

**FIGURE 2:**
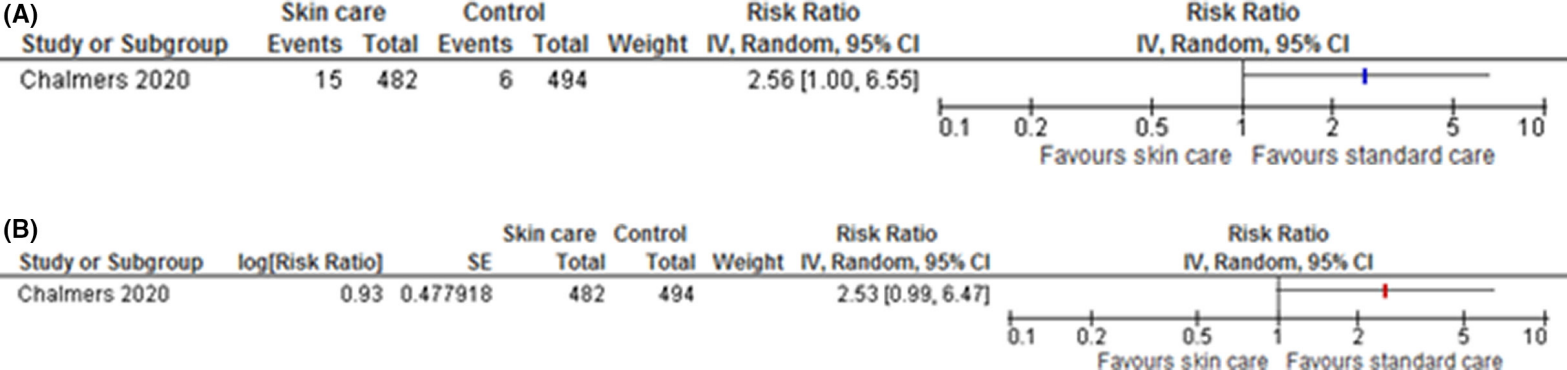
Food allergy IPD meta‐analysis forest plot versus aggregate data meta‐analysis forest plot. (A) Aggregate meta‐analysis—Food allergy confirmed by oral food challenge. (B) IPD meta‐analysis—Food allergy confirmed by oral food challenge

The search identified 33 eligible RCTs, with 25,827 participants of which 11 (5217 participants) had outcomes qualifying for inclusion in one or more meta‐analysis (see Figure S1). Of these 11 studies, 10 (5163 participants) were included in aggregate meta‐analysis. One trial, identified as completed via its trial registry record, had not yet been analysed or reported when the review was carried out^13^ so could not be included in the aggregate data meta‐analysis. This trial could be included in the previously conducted IPD meta‐analysis, which included 10 studies (5154 participants). In the IPD meta‐analysis, all 11 studies eligible for meta‐analysis were contacted and asked to provide IPD; one study did not respond nor provide IPD, Migacheva 2018.^14^ This study was not included in the primary IPD analysis; but was included in a sensitivity analysis which combined IPD with aggregate data. Figures 1, 2 and Table 1 show that for most outcomes, IPD enabled a greater number of studies to be included within pooled estimates.

**TABLE 1:** IPD versus aggregate meta‐analysis results for secondary outcomes

Secondary Outcome	IPD Meta‐analysis^a^		Aggregate meta‐analysis^b^	
	Effect estimate [95% CI]	Number of studies (I^2^)	Effect estimate [95% CI]	Number of studies (I^2^)
Adverse event: skin infection	1.33 [1.01, 1.75]	6 (0%)	1.31 [0.99, 1.75]	3 (0%)
Adverse event: stinging or allergic reaction to moisturizers	2.24 [0.67, 7.43]	4 (0%)	2.13 [0.43, 10.46]	1 (NA)
Adverse event: slippage accidents	1.42 [0.67, 2.99]	4 (0%)	1.36 [0.63, 2.94]	3, (0%)
Serious Adverse Events	1.80 [0.45, 7.18]	3 (51%)	0.62 [0.23, 1.70]	1 (NA)
Clinician‐assessed eczema severity (clear/mid vs. moderate/severe/very severe)	0.92 [0.37 to 2.27]	1^c^ (NA)	1.00 [0.42, 2.37]	2 (0%)
Clinician‐assessed eczema severity (standardized mean difference)	−0.02 [−0.17, 0.12]	3 (7%)	Not possible^e^	
POEM	1.17 [0.82, 1.67)	1 (NA)	1.18 [0.82, 1.68]^f^	1 (NA)
POEM (mean difference)	0.07 [−0.38, 0.52]	1 (NA)	Not possible	
Time to onset of eczema	0.86 [0.65, 1.14]	9 (53%)	0.59 [0.44, 0.80]	2 (0%)
Parent report of immediate (<2 hours) reaction to known common food allergen	1.27 [1.00, 1.61]	1 (NA)^d^	1.28 [1.00, 1.63]^f^	1 (NA)
Allergic sensitization to common foods or inhalants at 1–3 years	1.09 [0.72, 1.66]	2 (24%)	1.17 [0.89, 1.53]	2 (0%)
Allergic sensitization to common foods at 1–3 years	0.86 [0.28, 2.69]	2 (70%)	0.93 [0.34, 2.56]	2 (61%)
Allergic sensitization to milk at 1–3 years	1.16 [0.55, 2.43]	2 (0%)	1.18 [0.57, 2.49]	2 (0%)
Allergic sensitization to egg at 1–3 years	0.75 [0.18, 3.08]	2 (71%)	0.84 [0.25, 2.90]	2 (63%)
Allergic sensitization to peanut at 1–3 years	1.03 [0.53, 2.01]	2 (2%)	1.04 [0.54, 2.00]	2 (1%)
Allergic sensitization to inhalants at 1–3 years	1.09 [0.76, 1.57]	2 (0%)	1.09 [0.75, 1.58]	2 (0%)

^a^ Pooled estimates are unadjusted for AEs. For all other outcomes are adjusted for sex and family history of atopy following IPD meta‐analysis SAP.

^b^ Pooled estimated unadjusted unless otherwise indicated (for POEM and parental report of immediate reaction).

^c^ 1 study reported 0 events (NCT103376243, no aggregate data available) and a second study reported 1 moderate to very severe event in intervention arm (Lowe 2018) but was not included in IPD meta‐analysis as stage 1 analysis model did not converge.

^d^ A second study reported 0 events in IPD meta‐analysis (NCT103376243, no aggregate data available).

^e^ One trial only reported data in categories as clear/mild/moderate/severe/very severe, another median/IQR only.

^f^ Adjusted RR available from one study (centre, no. immediate family famers with atopic disease; 1, 2 or 2+) used (BEEP).

### 3.2 Aggregate versus IPD meta‐analysis for eczema

We extracted unadjusted aggregate data on the primary eczema outcome for 7 trials (3089 participants) which differed to the 7 trials (3075 participants) in the IPD meta‐analysis. Unlike the IPD meta‐analysis, study NCT03376243 could not be included as no aggregate data were available, but Migacheva 2018^15^ had aggregate data on eczema and was included. The unadjusted trial treatment estimates naturally differed slightly to the results used in the IPD meta‐analysis which were adjusted for sex and family history of atopy. In addition, for one trial, IPD enabled us to include 156 in the analysis and use the preferred, more robust, outcome of eczema by 2 years as diagnosed by a physician only (rather than diagnosis by a physician or a parental report) following the pre‐specified hierarchy of eczema diagnosis. Collected data on eczema from one participant had been excluded from the published analysis as they had incomplete baseline data on parental atopy, which was a covariate in the trials original published analysis. In another trial IPD revealed 86 cases of eczema as diagnosed by UKWP out of a denominator of 455, which differed slightly to the figures reported in the study authors publication of 86/459.

The pooled result for eczema was similar between analyses, IPD: adjusted RR 1.03, 95% CI [0.81, 1.31] (7 studies, I^2^ = 41%), aggregate: unadjusted RR 1.01 95% CI [0.77, 1.33] (7 studies, I^2^ = 53%). But, there was a slight increase in precision (95% CI width 11% narrower) and reduction of statistical heterogeneity in the IPD meta‐analysis. Moreover, in IPD meta‐analysis the statistical heterogeneity (41%) could be entirely explained by one trial with a RR favouring standard care (Skjerven). When this trial was excluded in IPD meta‐analysis the I^2^ reduced to 0. This may be explained by the intervention type (bathing with oil and moisturizer applied to the face only) and/or timing of intervention initiation (initiated from 2 weeks) in the Skjerven trial. In contrast in the aggregate meta‐analysis Skjerven did not alone explain the heterogeneity. After excluding Skjerven from the aggregate data, the pooled adjusted RR was 0.93, 95% CI [0.72, 1.20] I^2^ = 31%. Sensitivity analysis results using adjusted aggregate data where available were similar to primary analysis (see Table S1).

Results of aggregate data and IPD subgroup analyses (see Table S2) were similar, but also with reduced statistical heterogeneity in IPD meta‐analysis.

### 3.3 Aggregate versus IPD meta‐analysis for food allergy

One trial (Chalmers 2020) collected data on food allergy as confirmed by oral food challenge (Figure 2). A total of 15/482 confirmed food allergy cases were identified in the intervention group versus 6/494 in the control group in both IPD and aggregate data analysis. The aggregate data analysis resulted in an unadjusted RR = 2.56, 95% CI [1.00, 6.55]; The lower limit of the 95%CI included no difference (RR = 1). The IPD analysis resulted in an adjusted RR = 2.53, 95% CI [0.99, 6.47]. The use of adjustment in the IPD meta‐analysis provides a more powerful analysis, and resulted in a lower limit for the 95%CI which spanned down to 0.99, including no difference.

### 3.4 Aggregate versus IPD meta‐analysis for secondary outcomes

**FIGURE 3:**
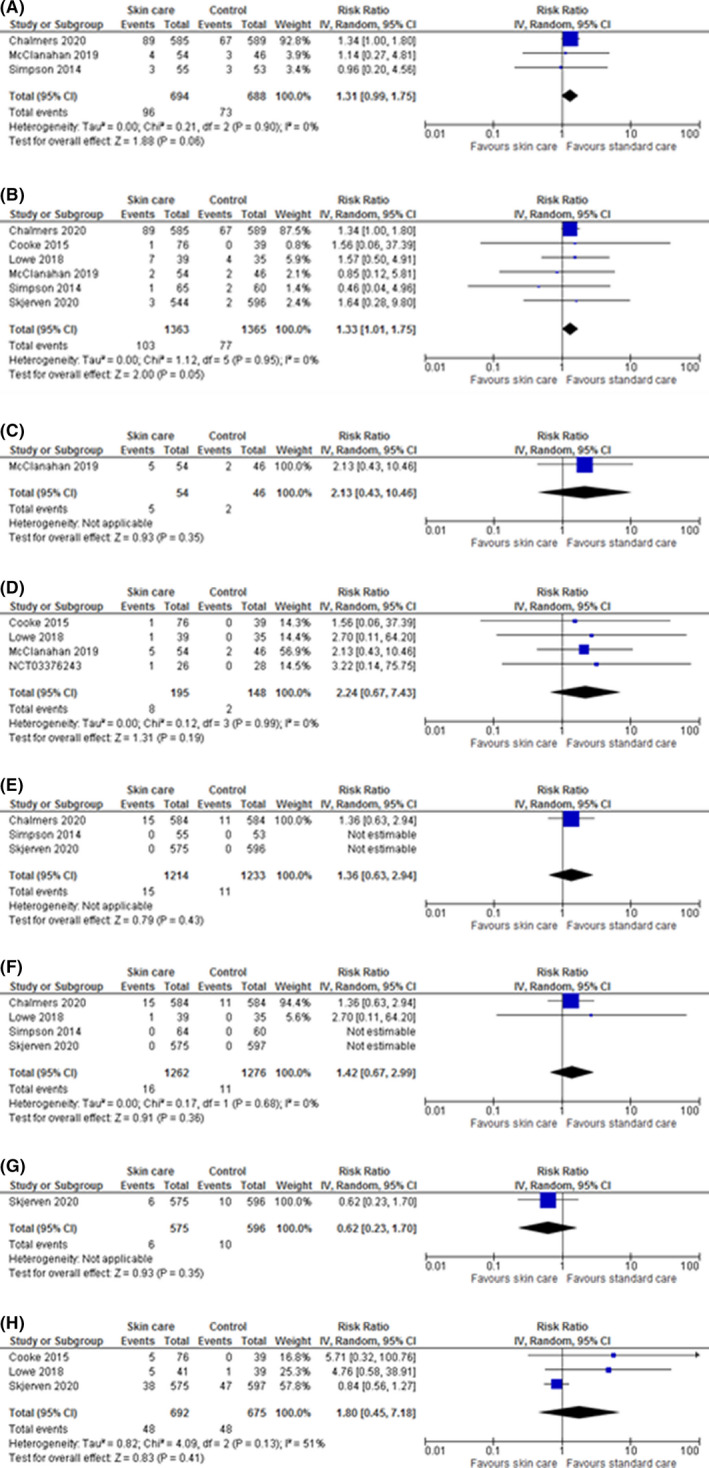
Aggregate versus IPD meta‐analysis results for Adverse Events. (A) Skin infection—aggregate meta‐analysis. (B) Skin infection—IPD meta‐analysis. (C) Stinging or allergic reactions—aggregate meta‐analysis. (D) Stinging or allergic reactions—IPD meta‐analysis. (E) Slippage—aggregate meta‐analysis. *There was one slippage in the skin care +early food introduction combined group in the Skjerven trial not eligible for meta‐analysis. (F) Slippage—IPD meta‐analysis. *There was one slippage in the skin care +early food introduction combined group in the Skjerven trial not eligible for meta‐analysis. (G) SAE—aggregate meta‐analysis. (H) SAE—IPD meta‐analysis

Aggregate data on skin infections were available for three studies (1382 participants) giving a pooled unadjusted RR 1.31, 95%CI [0.99, 1.75]. The lower limit of the 95%CI for the pooled result spans down to 0.99, including no difference. In IPD meta‐analysis, six trials (2728 participants) provided data and gave a pooled unadjusted RR of 1.33, 95% CI [1.01, 1.75]. The lower limit of the 95%CI for the pooled estimate excluded 1 giving a slightly more notable signal of harm, built upon a greater volume of participant data. A greater number of studies could also be included in the IPD analyses of stinging or application site reactions, slippage accidents and SAEs (Figure 3). IPD revealed not all SAEs had been reported in the trial publication for one trial. SAEs were only reported for participants who had had two or more SAEs; this information was not provided alongside published results.

Differences between IPD and aggregate meta‐analyses for other secondary outcomes are reported in Table 1 and were typically small. Only two trials (1163 participants) reported aggregate data on time to eczema giving a pooled unadjusted HR 0.59, 95% CI [0.44, 0.80]. Using the IPD, we could calculate time to eczema in a consistent manner across nine trials (3349 participants) using obtained visit dates and eczema outcomes, pooled adjusted HR 0.86, 95% CI [0.65, 1.14].

### 3.5 Analyses only possible using IPD

We could not conduct participant‐level treatment interaction meta‐analyses using aggregate data and were limited to individual trial reports. One trial (816 participants) previously reported the interaction effect for having 1 or 2 FLG mutations versus 0 on eczema (adjusted RR 1.20, 95% CI [0.70 to 2.09]). One other trial reported ‘No significant interaction effect of parental atopy was found with the skin intervention (*p* = 0·4)’. IPD meta‐analysis revealed no significant interactions between patients age at treatment initiation, FLG and family history and the treatment effect (see Table 2).

**TABLE 2:** Analyses only possible with IPD

Outcome	Effect estimate (RR)^a^ [95% CI]	No. studies (I^2^)
Sensitivity Analyses^b^		
Eczema after the intervention period (at 1 year or beyond ‐ up to 2 years)	1.06 [0.77, 1.47]	4 (45%)
Patient by treatment interactions^c^		
Eczema by 1–3 years for treatment initiation <4 days versus ≥4 days of age	1.05 [0.64, 1.73]	2 (0%)
Eczema by 6 months–3 years for treatment initiation <4 days versus ≥4 days of age	1.59 [0.56, 4.51]	3 (55%)
Eczema by 1–3 years by *FLG* genotype (0 mutations versus 1/2 mutations)	1.22 [0.71, 2.11]^d^	1 (NA)
Eczema by 6months–3 years by *FLG* genotype (0 mutations versus 1/2 mutations)	1.03 [0.42, 2.51]	2 (16%)
Eczema by 1–3 years by ≥1 first degree relative with history of allergic disease	0.95 [0.35, 2.61]	3 (0%)
Food allergy by 1–3 years for treatment initiation <4 days versus ≥4 days of age	0.51 [0.07, 3.53]	1 (NA)
Primary CACE analysis		
Eczema by 1–3 years for use over intervention period ≥3 days a week	0.65 [0.29, 1.45]	3 (0%)
Food allergy by 1–3 years for use over intervention period ≥3 days a week	31.19 [0.43, 2236.62] ‡	1 (NA)
CACE sensitivity analysis ‐ Eczema by 1–3 years		
Use over intervention period ≥5 days a week	0.74 [0.26, 2.09]	2 (0%)
Use over intervention period 7 days a week	0.78 [0.23, 2.71]	3 (0%)
Use over first 3 months ≥3 days a week	1.02 [0.79, 1.31]	2 (0%)
Use over first 3 months ≥5 days a week	0.84 [0.46, 1.52]	2 (0%)
Use over first 3 months 7 days a week	0.83 [0.34, 2.03]	3 (0%)
ITT for studies included in CACE for use over intervention period ≥3 days a week	0.93 [0.77, 1.12]	3 (0%)
ITT for studies included in CACE for use over intervention period ≥5 days a week	0.95 [0.79, 1.15]	2 (0%)
ITT for studies included in CACE for use over intervention period 7 days a week	0.95 [0.79, 1.15]	3 (0%)
ITT for studies included in CACE for use over first 3 months ≥3 days a week	0.95 [0.79, 1.15]	2 (0%)
ITT for studies included in CACE for use over first 3 months ≥5 days a week	0.95 [0.79, 1.15]	2 (0%)
ITT for studies included in CACE for use over first 3 months 7 days a week	0.95 [0.79, 1.15]	3 (0%)
CACE sensitivity analysis—Food allergy by 1–3 years^e^		
Use over intervention period ≥5 days a week	47.47 [0.09, 24643.91]^e^	1 (NA)
Use over intervention period 7 days a week	125.21 [0.00, 3150317.50]^e^	1 (NA)
Use over first 3 months ≥3 days a week	7.39 [0.79, 69.02]^e^	1 (NA)
Use over first 3 months ≥5 days a week	8.08 [0.56, 116.23]^e^	1 (NA)
Use over first 3 months 7 days a week	19.11 [0.11, 3310.01]^e^	1 (NA)

^a^ All pooled effect estimates are Risk Ratios adjusted for sex and parental atopy.

^b^ Intervention effect quantifies the risk of the eczema for skin care intervention use versus standard care or no skin care intervention.

^c^ Patient by treatment interactions represent the Relative Risk of the outcome (eczema or food allergy) for skin care intervention versus standard care or no skin care intervention for the associated characteristic [relative to absence of that characteristic].

^d^ For 1 additional study the interaction effect was not estimable as all standard care participants with *FLG* mutations (1 or 2 mutations) had eczema, i.e. the interaction predicts eczema perfectly. In the standard care group 5/22 (0 mutations) and 1/1 (1 or 2 mutations) had eczema. In the skin care intervention group 3/21 0 mutations) and 1/3 (1 or 2 mutations). ITT = Intention‐to‐treat. CACE estimates represent the relative risk of the outcome (eczema or food allergy) for skin care intervention use versus standard care or no skin care intervention among those who would comply with the allocated interventions.

^e^ CACE estimates for the one trial reporting food allergy were accompanied by wide 95% CI’s suggesting a problem with the estimation procedure.

Meta‐analysis for the Complier Average Causal Effect (CACE) was not possible using aggregate data. Only one trial previously reported the CACE for eczema as an adjusted OR (adjusted for centre and parental atopy) for intervention use ≥3 days/week over the first 3 months, adjusted OR = 0.88 (0.50–1.56). IPD enabled computation of compliance indicators, using consistent thresholds across trials supplying compliance data, and conduction of additional CACE analysis that was not previously performed. The pooled CACE estimates did not provide evidence that treatment adherence impacted risk of eczema (see Table 2).

### 3.6 Risk of bias

IPD beneficially impacted risk of bias assessments (see Table 3). For the co‐primary eczema outcome, 3/7 studies were judged low risk of bias (4/7 some concerns of risk of bias) in the IPD meta‐analysis, compared to only 1/7 in aggregate meta‐analysis (6/7 some concerns of risk of bias). This was due to: (1) IPD enabled additional missing data sensitivity analysis that had not previously been reported, (2) In IPD meta‐analysis, the risk of bias for the selection of the reported results was low as a pre‐specified SAP, finalized before any unblinded data was available, was used for meta‐analysis. In the aggregate meta‐analysis, three studies did not provide adequate pre‐specified information on a trial registry or whether a pre‐specified SAP was followed, resulting in some concerns on this domain.

**TABLE 3:** Risk of bias assessments for aggregate data and IPD meta‐analysis

Outcome	Study	Risk of Bias domain rating						Explanation of difference between aggregate data and IPD
		1	2	3	4	5	Overall	
Eczema by 1 to 2 years	Chalmers 2020	Low	Low	Low	Low	Low	Low	
	Dissanayake 2019	Low	Low	Some concerns	Low	Some concerns	Some concerns	No missing data sensitivity analysis in paper. Trial registry unavailable and no information on whether a pre‐specified SAP followed (unlikely that multiple eligible outcomes/analysed).
	Lowe 2018	Low	Low	Some concerns	Low	Low	Some concerns	No missing data sensitivity analysis in paper. Trial registry pre‐specified eczema measurement using UKWP criteria and time point.
	Mc Clanahan 2019	Low	Low	Some concerns	Low	Some concerns	Some concerns	Trial registry indicates atopic dermatitis diagnosed by investigator at 12 months, but not mention of UKWP and no other information on whether a pre‐specified SAP followed (unlikely that multiple eligible outcomes/analysed).
	Migacheva 2018	Some concerns	Some concerns	Some concerns	Some concerns	Some concerns	Some concerns	
	Skjerven 2020	Low	Low	Some concerns	Low	Low	Some concerns	
	Yonezawa 2018	Low	Low	Some concerns	Low	Some concerns	Some concerns	No missing data sensitivity analysis in paper. Trial registry only pre‐specified outcomes up to 3 months and no other information on whether a pre‐specified SAP followed (unlikely that multiple eligible outcomes/analysed).
Food allergy (oral food challenge) by 1 to 2 years	Chalmers 2020	Low	Low	High	Low	Low	High	
Slippages (over the intervention period)	Chalmers 2020	Low	Low	Low	Some concerns	Low	Some concerns	
	Simpson 2014	Low	Low	Low	Some concerns	Low	Some concerns	Trial registry pre‐specified incidence of emollient‐related adverse events.
	Skjerven 2020	Low	Low	Low	Some concerns	Low	Some concerns	
Skin infection (over the intervention period)	Chalmers 2020	Low	Low	Low	Some concerns	Low	Some concerns	
	Mc Clanahan 2019	Low	Low	Low	Some concerns	Some concerns	Some concerns	Outcome not pre‐specified on trial registry and no other information on whether a pre‐specified SAP followed (unlikely that multiple eligible outcomes/analyses)
	Simpson 2014	Low	Low	Low	Some concerns	Low	Some concerns	Trial registry pre‐specified incidence of emollient‐related adverse events.
Stinging/allergic reactions to moisturizers (over the intervention period)	Mc Clanahan 2019	Low	Low	Low	Some concerns	Some concerns	Some concerns	Outcome not pre‐specified on trial registry and no other information on whether a pre‐specified SAP followed (unlikely that multiple eligible outcomes/analyses)
Time to onset of eczema	Horimukai 2014	Low	Low	Low	Low	Some concerns	Some concerns	Outcome not pre‐specified on trial registry and no other information on whether a pre‐specified SAP followed (unlikely that multiple eligible outcomes/analyses)
	Skjerven 2020	Low	Low	Some concerns	Low	Low	Some concerns	
Parent report of immediate reaction to common food allergen (2 years)	Chalmers 2020	Low	Low	Some concerns	Low	Low	Some concerns	No missing data sensitivity analysis in paper
Allergic sensitization to a food allergen (1 to 2 years)	Chalmers 2020	Low	Low	Some concerns	Low	Low	Some concerns	No missing data sensitivity analysis in paper
	Lowe 2018	Low	Low	Some concerns	Low	Low	Some concerns	No missing data sensitivity analysis in paper. Trial registry pre‐specified outcome measurement via skin prick tests (defined as a wheal of 3mm or greater).

Note

Shading indicates difference between IPD and aggregate ROB2 rating. In all cases of differences these were ‘Low’ with IPD but ‘Some concerns’ using aggregate data only. No aggregate data meta‐analysis for IgE‐mediated food allergy (oral food challenge) by 1 to 2 years.

### 3.7 Quality of the evidence

The quality of the evidence for the aggregate meta‐analysis is summarized in Table 4 and for the IPD analysis in Table 5. For the co‐primary eczema outcome, due to increased unexplained statistical heterogeneity and more trials rated as some concerns of risk of bias in the aggregate analysis, there is low certainty evidence; in IPD meta‐analysis there was moderate certainty evidence. For two other secondary outcomes, the certainty of evidence is lower by one grade in the aggregate meta‐analysis versus IPD, with reasons given in Table 3. Table 6 summarizes the impact of these differences for making judgments on the review outcomes.

**TABLE 4:** Summary of findings and quality of evidence in the aggregate meta‐analysis

Outcome	Assumed risk	Corresponding risk		No. participants (studies)	Quality of the evidence (GRADE)	Comments	Differences for aggregate data v IPD meta‐analysis and explanation of grading
	Standard care	Skin care intervention	Relative effect (95% CI)				
Eczema diagnosis by 1 to 2 years	150 per 1000	152 per 1000 (112 to 200)	RR 1.01 (0.77 to 1.33)	3089 (7)	Low	In sensitivity analysis that included studies that measured eczema using Hanifin and Rajka, or UK Working Party methods only, total *N* = 2874(5), the pooled treatment effect for eczema by 1 to 2 years was RR 1.05, 95% CI 0.79 to 1.40. Only one study was rated as low risk of bias only, total *N* = 1210 (1), RR 0.95, 95% CI 0.78 to 1.16.	Downgraded from moderate (IPD) to low (aggregate data). In aggregate meta‐analysis: Downgraded one as majority of studies have some concerns of risk of bias and downgraded one as statistical heterogeneity across trials is unexplained. In IPD meta‐analysis: Downgraded one level for heterogeneity driven by one trial with a different (bathing) intervention to the other trials.
IgE‐mediated food allergy (oral food challenge) by 1 to 2 years	50 per 1000	128 per 1000 (50 to 328)	RR 2.56 [1.00 to 6.55]	976 (1)	Very low	In a sensitivity analysis that examined IgE‐mediated food allergy as measured by oral food challenge or based upon a panel assessment of clinical history and/or allergic sensitization by 1 to 2 years, total *N* = 1115 (1), was RR = 1.47, 95% CI 0.93 to 2.33. For parent report of a doctor diagnosis of food allergy at 1 to 2 years, total *N* = 1602 (3), and the pooled treatment effect was RR 1.07, 95% CI 0.83 to 1.37. No low risk of bias sensitivity analysis was possible	Same grading as IPD. Downgraded one level for overall high risk of bias due to missing data (30%), and two levels for imprecision due to small numbers of events from a single study, with wide confidence intervals, which include both a harmful effect and no effect.
Slippages (over the intervention period)	20 per 1000	28 per 1000 (13 to 59)	RR 1.36 [0.63 to 2.94]	2447 (3)	Low		Same grading as IPD. Downgraded by two levels for imprecision due to small numbers of events, with wide confidence intervals, which include both a harmful effect and a beneficial effect.
Skin infection (over the intervention period)	50 per 1000	66 per 1000 (66 to 88)	RR 1.31, 95%CI [0.99 to 1.75]	1382 (3)	Moderate		Same grading as IPD. In aggregate meta‐analysis: Downgraded by one level for imprecision due to small numbers of events, with wide confidence intervals, which include both a harmful effect and a beneficial effect (3 trials). In IPD meta‐analysis: Downgraded by one level for imprecision due to wide confidence intervals, which include both a harmful effect and no effect (6 trials)
Stinging/allergic reactions to moisturizers (over the intervention period)	40 per 1000	86 per 1000 (18 to 419)	RR 2.13, 95% [0.43 to 10.46]	100 (1)	very low		Downgraded from low (IPD) to very low (aggregate). In aggregate meta‐analysis: Downgraded one level for some concerns of risk of bias due to potential selective reporting, and two levels for imprecision due to small numbers of events from a single study, with wide confidence intervals, which include both a harmful effect and no effect. In IPD meta‐analysis: Downgraded by two levels for imprecision due to small numbers of events, with wide confidence intervals, which include both a harmful effect and a beneficial effect (4 trials).
Time to onset of eczema	24 months	40.7 months (30 to 54.5)	HR 0.59, 95% CI [0.44 to 0.80]	1163 (2)	low		Downgraded from moderate (IPD) to low (aggregate). In aggregate meta‐analysis: Downgraded one level for some concerns of risk of bias in the two included trials due to missing data or potential selective reporting, and one level for clinical heterogeneity (one trial had only 32 week follow‐up versus the second had 2 years follow‐up). Small numbers of events from two studies, with wide confidence intervals, although all estimates represent a clinically meaningful increase in time to onset of eczema. In IPD meta‐analysis: Downgraded one level for heterogeneity driven by more than one trial, for which review authors were unable to identify a plausible explanation.
Parent report of immediate reaction to common food allergen (at 2 years)	160 per 1000	205 per 1000 (160 to 261)	RR 1.28, 95%CI [1.00 to 1.63]	1171 (1)	low		Same grading as IPD. In aggregate meta‐analysis: Downgraded two levels for imprecision due to small numbers of events from a single study, with wide confidence intervals, which include both a harmful effect and no effect. In IPD meta‐analysis: Downgraded two levels for imprecision due to small numbers of events from a single study, with wide confidence intervals, which include both a harmful effect and no effect.
Allergic sensitization to a food allergen (at 1 to 2 years)	90 per 1000	84 per 1000 (31 to 231)	0.93 95% CI [0.34 to 2.56]	1055 (2)	very low		Same as IPD meta‐analysis. Downgraded one level for heterogeneity, for which the review authors were unable to identify a plausible explanation, and two levels for imprecision due to wide confidence intervals, which include both a harmful and a beneficial effect and some concern of risk of bias due to missing data across both included trials.

Note

All estimated are unadjusted. Shading indicates one grade lower than IPD meta‐analysis.

**TABLE 5:** Summary of findings and quality of evidence in the IPD meta‐analysis

Outcome	Assumed risk	Corresponding risk		No. participants (studies)	Quality of the evidence (GRADE)	Comments
	Standard care	Skin care intervention	Relative effect (95% CI)			
Eczema diagnosis by 1 to 2 years	150 per 1000	155 per 1000 (122 to 197)	RR 1.03 (0.81 to 1.31)	3075 (7)	MODERATE^a^	In sensitivity analysis that included studies that measured eczema using Hanifin and Rajka, or UK Working Party methods only, total *N* = 2919(6), the pooled treatment effect for eczema by 1 to 2 years was RR 1.02, 95% CI 0.78 to 1.34. In a separate sensitivity analysis including studies rated as low risk of bias only, total *N* = 1739(3), the pooled treatment effect for eczema by 1 to 2 years was RR 0.97, 95% CI 0.81 to 1.17
IgE‐mediated food allergy (oral food challenge) by 1 to 2 years	50 per 1000	127 per 1000 (50 to 335)	RR 2.53 (0.99 to 6.47)	976 (1)	VERY LOW^b^	In a sensitivity analysis that examined IgE‐mediated food allergy as measured by oral food challenge or based upon a panel assessment of clinical history and/or allergic sensitization by 1 to 2 years, total *N* = 1115 (1), was RR = 1.46, 95% CI 0.91 to 2.34. For parent report of a doctor diagnosis of food allergy at 1 to 2 years, total *N* = 1614 (3), and the pooled treatment effect was RR 1.02, 95% CI 0.80 to 1.31. No low risk of bias sensitivity analysis was possible
Slippages (over the intervention period)	20 per 1000	29 per 1000 (14 to 87)	RR 1.42 (0.67 to 2.99)	2538 (4)	LOW^c^	‐
Skin infection (over the intervention period)	50 per 1000	67 per 1000 (51 to 88)	RR 1.33 (1.01 to 1.75)	2728 (6)	MODERATE^d^	‐
Stinging/allergic reactions to moisturizers (over the intervention period)	40 per 1000	90 per 1000 (27 to 298)	RR 2.24 (0.67 to 7.43)	343 (4)	LOW^c^	‐
Time to onset of eczema	24 months	27.9 months (21.1 to 36.9 months)	HR 0.86 (0.65 to 1.14)	3349 (9)	MODERATE^e^	‐
Parent report of immediate reaction to common food allergen (at 2 years)	160 per 1000	204 per 1000 (160 to 258)	RR 1.27 (1.00 to 1.61)	1171 (1)	LOW^f^	‐
Allergic sensitisation to a food allergen (at 1 to 2 years)	90 per 1000	78 per 1000 (26 to 242)	RR 0.86 (0.28 to 2.69)	1055 (2)	VERY LOW^g^	

Note

This table is a replicate of the summary of findings table reported in, Kelleher MM, Cro S, Cornelius V, Lodrup Carlsen KC, Skjerven HO, Rehbinder EM, Lowe AJ, Dissanayake E, Shimojo N, Yonezawa K, Ohya Y, Yamamoto‐Hanada K, Morita K, Axon E, Surber C, Cork M, Cooke A, Tran L, Van Vogt E, Schmitt J, Weidinger S, McClanahan D, Simpson E, Duley L, Askie LM, Chalmers JR, Williams HC, Boyle RJ. Skin care interventions in infants for preventing eczema and food allergy. Cochrane Database of Systematic Reviews 2021, Issue 2. Art. No.: CD013534. 10.1002/14651858.CD013534.pub2. Accessed 16 May 2021

^a^ Downgraded one level for heterogeneity driven by one trial contributing 21.8% of the weight of the analysis, for which the review authors were unable to identify a plausible explanation.

^b^ Downgraded one level for overall high risk of bias due to missing data (29%), and two levels for imprecision due to small numbers of events from a single study, with wide confidence intervals, which include both a harmful effect and no effect.

^c^ Downgraded by two levels for imprecision due to small numbers of events, with wide confidence intervals, which include both a harmful effect and a beneficial effect.

^d^ Downgraded by one level for imprecision due to wide confidence intervals, which include both a harmful effect and no effect.

^e^ Downgraded one level for heterogeneity driven by more than one trial, for which review authors were unable to identify a plausible explanation.

^f^ Downgraded two levels for imprecision due to small numbers of events from a single study, with wide confidence intervals, which include both a harmful effect and no effect.

^g^ Downgraded one level for heterogeneity, for which the review authors were unable to identify a plausible explanation, and two levels for imprecision due to wide confidence intervals, which include both a harmful and a beneficial effect.

**TABLE 6:** Statements of effects from IPD meta‐analysis versus aggregate data meta‐analysis

Outcome	Summary of IPD result	Summary of aggregate data result
Eczema by 1 to 2 years	Skin care interventions during infancy probably do not change risk of eczema by one to two years of age	Skin care interventions during infancy may not change risk of eczema by one to two years of age
IgE‐mediated food allergy (oral food challenge) by 1 to 2 years	It is unclear whether skin care interventions during infancy change risk of IgE‐mediated food allergy by one to two years of age	It is unclear whether skin care interventions during infancy change risk of IgE‐mediated food allergy by one to two years of age
Slippages (over the intervention period)	Skin care intervention may increase risk of infant slippage over the intervention period	Skin care intervention may increase risk of infant slippage over the intervention period
Skin infection (over the intervention period)	Skin care interventions during infancy probably increase risk of skin infection over the intervention period	Skin care interventions during infancy probably increase risk of skin infection over the intervention period
Stinging/allergic reactions to moisturizers (over the intervention period)	Skin care intervention may increase risk of stinging/allergic reactions to moisturizers	It is unclear whether skin care interventions during infancy affect the risk of stinging/allergic reactions to moisturizers
Time to onset of eczema	Skin care interventions during infancy probably do not change time to onset of eczema	Skin care interventions during infancy may not change time to onset of eczema
Parent report of immediate reaction to common food allergen at 2 years	Skin care interventions during infancy may slightly increase risk of parent report of immediate reaction to a common food allergen at two years	Skin care interventions during infancy may slightly increase risk of parent report of immediate reaction to a common food allergen at two years
Allergic sensitisation to a food allergen at 1 to 2 years	It is unclear whether skin care interventions during infancy change allergic sensitisation by one to two years of age	It is unclear whether skin care interventions during infancy change allergic sensitisation by one to two years of age

Note

Shading indicates a difference between the IPD and aggregate meta‐analysis.

### 3.8 Resources required for the IPD meta‐analysis

IPD meta‐analysis can be retrospective, where previously published trial data is shared by collaborators, or prospective where collaborating groups agree prior to trial publication to share data. Our IPD meta‐analysis included prospective IPD meta‐analysis, though some of the smaller pilot studies had been published previously. This necessitated open, trusting relationships with collaborators who shared data prior to its publication, which took effort and time, making it slower and more costly than retrospective IPD meta‐analysis.

IPD analysis also required data sharing agreements between institutions, as de‐identified data is shared by the collaborating groups.

The IPD meta‐analysis needed increased time for the statistical analysis (50% FTE statistician for 2 years), and costs for project management which included searching, data extraction, data sharing agreements, data transfer and data cleaning (100% full time equivalent (FTE) for 2 years), collaborator meetings and international travel beyond the typical costs for an aggregate systematic review and meta‐analysis.

## 4 DISCUSSION

### 4.1 Main Findings

In this evaluation, we explored differences in outcomes from a Cochrane systematic review depending on the approach to data collection and analysis. We compared IPD with aggregate data meta‐analysis. We found that while risk ratios for primary outcomes were very similar, GRADE certainty of evidence ratings, adverse events analyses, subgroup and adherence analyses were all either significantly different with IPD or only possible with IPD compared with aggregate data meta‐analysis. IPD allowed for a better understanding of the primary eczema outcome and explanation of the statistical heterogeneity. Overall, use of IPD allowed a conclusion that the skincare interventions studied *probably do not* affect eczema risk; compared with a conclusion that they *may not* affect eczema risk when using aggregate data meta‐analysis.

There were significant differences in findings for secondary outcomes, with IPD generally able to offer more reliable and precise information on important potential harms such as increased skin infections with emollients. A key benefit of IPD meta‐analysis realized here was better powered analysis of specific adverse events. The signals of harm were not so clear without the IPD. Publications do not always adequately report all the information on specific events^16^ as we found here. Publications typically only report a subset of AEs experienced by participants, relying on arbitrary rule‐based approaches to select events for inclusion.^13^, ^17^ For example, Skjerven et al.^18^ chose to only report SAEs experienced by two or more participants. Inconsistent selection criteria across trials hampers aggregate meta‐analysis but with access to IPD, treatment effect estimates for AEs are more reliable. One explanation of this is that journal word count is limited. The analysis shown here demonstrated an improved understanding of adverse events with access to IPD. IPD may therefore be particularly useful in other settings when specific adverse events are of interest.

Overall the IPD meta‐analysis was more resourceful in terms of time, research staff and associated financial costs and was only possible due to trusting relationships across a large collaborative group of trialists who were willing to share anonymized data; some before the primary publication of their results. Barriers to IPD meta‐analysis may include an unwillingness to share data. To facilitate data sharing within this review, data sharing agreements between each data provider and the SCiPAD research group included the conditions for secure data transfer and holding and that any results were not to be published without approval from all data suppliers. Data suppliers could nominate up to three representatives to serve on the authorship group for the main IPD meta‐analysis.

The previously conducted IPD meta‐analysis was performed using a two‐stage analytical approach. This approach was originally chosen since individual study effects are immediately available for examination in forest plots alongside pooled results and it enables IPD and aggregate data for any trials not providing IPD to be readily combined in the same analysis (a sensitivity analysis conducted within the original Cochrane review combined IPD and aggregate data from one trial that did not supply IPD). A two‐stage approach also proved valuable as one trial providing IPD gave access to their data in an online secure platform and it could not be exported to be directly combined with the other trial data sets, which a one‐stage analytical approach requires. Previous research indicates that in most cases similar results will be obtained from a one‐stage and two‐stage IPD meta‐analysis.^19^, ^20^ Where differences are reported this is because (i) researchers have knowingly or unknowingly made different modelling assumptions and/or (ii) used different estimation methods for deriving point estimates or confidence intervals. Where assumptions and estimations do not vary the two approaches will give similar results, enabling analysts to choose the most convenient procedure.

### 4.2 How our study compares with others

The results of this case study demonstrate advantages of IPD meta‐analysis previously discussed by others.^2^, ^3^, ^4^ Using 18 cancer systematic reviews, Tierney demonstrated how when the total number of participants or events (‘absolute information size’) and the proportion of eligible participants or events available from aggregate data relative to the IPD (‘relative information size’) are both large, as in this review, the results of an aggregate data‐meta analysis are likely to agree with those of an IPD meta‐analysis.^4^ Thus, they concluded that there may be little justification for collecting IPD, unless an intervention effect had been detected and more detailed analyses are required. Similarly, we did not find large differences in pooled treatment effects between IPD and aggregate data analyses. But the reduction in statistical heterogeneity in the IPD meta‐analysis improved the certainty in the finding for the primary outcome, for which all heterogeneity could be explained in the IPD analysis. Further, for one secondary harm outcome, although there was only a small difference in the pooled treatment effect sizes, the difference rendered the outcomes more notable in IPD analysis as the confidence interval excluded the null effect; this was not the case in the aggregate analysis, potentially leading to different conclusions by policy makers.

We identified a strong benefit of IPD analysis for exploring the harms of interventions. Publications do not always report complete adverse event information, but access to IPD on adverse events enables all events of interest to be included within evidence synthesis. Additionally, we have demonstrated how the ability to conduct more detailed analyses and target treatment estimands not calculated in included trials may also prove of value in settings where a significant treatment effect has not been detected to generate deeper insights on the impact of adherence.

### 4.3 Strengths and limitations of this study

The statistical analysis of the aggregate data performed here was conducted by the same researchers who performed the original IPD analysis. The authors who previously conducted the IPD risk of bias assessments went back to the papers to conduct risk of bias assessments for this aggregate analysis, enabling consistency in judgement. However, this could also be viewed as a weakness since they were already exposed to unique information from the IPD analysis and not blind to both bodies of data. The IPD and aggregate meta‐analysis results are not directly comparable, as we are not comparing like with like (adjusted versus unadjusted results). Therefore, it was not possible to fully separate the differences that are accounted for by aggregate versus IPD methods as opposed to the adjustment factors used consistently across study datasets in the IPD meta‐analysis (sex and family history of atopic disease). Adjustment for key prognostic factors was made in the IPD meta‐analysis^12^ to increase power in the analysis^21^; since all included studied were randomized trials, these factors were not expected to vary across intervention groups. In the aggregate data meta‐analysis, we only included published data and did not include any author correspondence, as we had already obtained IPD from trial authors. In the original Cochrane IPD analysis, IPD was available for the majority of eligible studies (88% for primary eczema outcome). A previous study reported only 43% of IPD meta‐analyses retrieved 80% or more relevant IPD which may thus limit the generalizability of this case study.^1^ However, as demonstrated in the original Cochrane review an IPD meta‐analysis is not limited to using IPD only; methods exist for combining IPD with aggregate data to minimize bias when IPD availability is lower.^22^, ^23^

## 5 CONCLUSION

In this evaluation of a Cochrane systematic review of skincare interventions for preventing eczema and food allergy, we found significant advantages to using an IPD meta‐analysis approach. IPD analysis resulted in higher certainty evidence, new adverse event information and enabled the exploration of treatment estimands not previously calculated by trialists, including those which quantify the impact of adherence and treatment interactions. Significant collaboration and cooperation between trialists and systematic reviewers is needed to achieve IPD meta‐analysis, but the process is likely to reduce future research waste by reducing uncertainty of findings.

## CONFLICTS OF INTEREST

HCW was director of the NIHR Health Technology Assessment (HTA) Programme until 1 October 2020, which is part of the NIHR that also supports the NIHR systematic reviews programme from which this work is funded; was also chief investigator of the BEEP study, which was funded by NIHR HTA and is included in this review; and has received funds (Nottingham) from the National Institute for Health Research (public funds) as a result of open competition. RJB has received payment for participating in advisory boards for DBV technologies, Prota therapeutics and ALK‐Abello, who develop allergy diagnostics or treatments; has received payment for designing a clinical trial for Dairy Goat Co‐operative; and has received payment for providing expert testimony in a class action related to an infant formula health claim. EVV, SC, VRC, LMA, RP and MK declared none.

## AUTHORS CONTRIBUTIONS

SC conceived of this impact evaluation. RJB and HW conceived of the meta‐analysis. SC and VC wrote the statistical analysis plan. RJB and MK contributed to the statistical analysis plan. SC and EVV obtained data for the aggregate data analysis, analysed and interpreted data. RJB and MK reviewed and commented on data analyses, did GRADE evaluations and drafted the summary of findings table. RJB, MK and SC conducted risk of bias assessments. LMA provided advice and expertize on conducting a prospective IPD meta‐analysis. EVV and SC wrote the first draft of the manuscript. EVV, SC, RJB and MK wrote the manuscript. All authors advised on data interpretation, critically reviewed and contributed to this manuscript.

## ETHICAL STATEMENT

Ethical approval was not required for this study which retrieved and synthesized data from already published studies. The IPD meta‐analysis was approved by the Imperial College London Research Ethics Committee on 18 May 2018 (reference 18IC4563). This was a prospectively planned individual patient data meta‐analysis, registered on Prospero in February 2017 (PROSPERO 2017 CRD42017056965).

## Supporting information

Supplementary Material

## Data Availability

The data analysed within this new aggregate data meta‐analysis is available from the corresponding author upon reasonable request.
