# Supplementary material for: Individual participant data meta‐analysis versus aggregate data meta‐analysis: A case study in eczema and food allergy prevention
Source: Clin Exp Allergy. 2022 Jan 10;52(5):628–45. doi: 10.1111/cea.14085 (PMC9302682; doi:10.1111/cea.14085)
Supplement: Supplementary file 1 — Supplementary Material [file CEA-52-628-s001.docx]

**Supplementary material**

Contents

[Figure S1 - PRISMA flow diagram 2](#_Toc85278812)

[Table S1 - Sensitivity IPD vs aggregate data meta-analysis 3](#_Toc85278813)

[Table S2 – Subgroup IPD vs aggregate data meta-analysis 3](#_Toc85278814)

# Figure S1 - PRISMA flow diagram

5230 additional records identified from trial registers

1816 records identified from the database search

22 studies excluded as did not measure one or more of the reviews co-primary or secondary outcomes

**Aggregate data meta-analysis:**

10 studies were included in one or more meta-analysis

**IPD meta-analysis:**

10 studies contributed IPD and 11* were included in one or more meta-analysis

150 studies (152 articles) Excluded due to wring study design (52), participants (79), intervention (6) or comparators (13)

11 studies (15 articles) either ongoing or not able to be classified

242 full-text articles assessed for eligibility

5891 records excluded based on titles and abstracts

6133 records after duplicates removed

33 studies (75 articles) included

*A sensitivity analysis for eczema included one trial that did not supply IPD but had aggregate data with the rest of the IPD data. In IPD meta-analysis it is possible to combine IPD with aggregate data results.

11 studies had one or more outcome qualifying for inclusion in one or more meta-analysis

Of the 7 trials included in the aggregate data meta-analysis, 2 also provided adjusted RR’s for eczema which were included in place of unadjusted estimates for these trials in an aggregate data sensitivity analysis. BEEP adjusted for recruitment site and number of first-degree relatives with atopic disease (stratification variables) and PEBBLES adjusted for so presence of siblings and forehead TEWL. No difference was found in the aggregate data sensitivity analysis (RR 1.01, 95%CI [0.77, 1.33] (7 studies, I^2^=53%)).

# Table S1 - Sensitivity IPD vs aggregate data meta-analysis

| Sensitivity Outcome | IPD | | Aggregate data | |
| --- | --- | --- | --- | --- |
|  | **Estimate [95% CI]** | **No. studies (I^2^)** | **Estimate [95% CI]** | **No. studies (I^2^)** |
| Eczema by 1-3 years (UKWP only) | 1.02 [0.78 , 1.34] | 6 (51%) | 1.05 [0.79, 1.4] | 5 (55%) |
| Eczema by 1-3 years (including data from all 4 arms of PreventADALL) | 1.03 [0.81 , 1.31] | 7 (41%) | 0.98 [0.81, 1.16] | 7 (20%) |
| Eczema by 1-3 years - low risk of bias in IPD meta-analysis† | 0.97 [0.81 , 1.17] | 3 (7%) | 0.99 [0.77, 1.26] | 3 (26%) |
| Eczema by 6 months -3 years | 0.89 [0.70 , 1.14] | 9 (55%) | 0.86 [0.67, 1.11] | 11 (58%) |
| Food allergy by 1-3 years (parent report of doctor diagnosis) | 1.02 [0.80 , 1.31] | 3 (0%) | 1.07 [0.83, 1.37] | 3 (0%) |

†In aggregate data meta-analysis only one study was rated at low risk of bias, N = 1210 (1),  RR 0.95, 95% CI 0.78 to 1.16.

## Table S2 – Subgroup IPD vs aggregate data meta-analysis

| Outcome | Subgroup | IPD | | Aggregate data | |
| --- | --- | --- | --- | --- | --- |
|  |  | **Estimate [95% CI]** | **No. studies (I^2^)** | **Estimate [95% CI]** | **No. studies (I^2^)** |
| Eczema by 1-3 years by intervention type | Basic emollients | 1.04 [0.66, 1.65] | 3 (75%) | 0.93 [0.61, 1.43] | 4 (70%) |
|  | Complex emollients | 1.01 [0.75, 1.37] | 4 (0%) | 1.10 [0.75, 1.60] | 3 (22%) |
|  | Total | 1.03 [0.81, 1.31] | 7 (41%) | 1.01 [0.77, 1.33] | 7 (53%) |
| Eczema by 1-3 years by prescribed intervention duration | ≤6 months | 1.01 [0.45, 2.27] | 1 (NA) | 1.31 [0.67, 2.56] | 1 (NA) |
|  | >6months | 1.02 [0.78, 1.34] | 6 (51%) | 0.97 [0.72, 1.31] | 6 (59%) |
|  | Total | 1.03 [0.81, 1.31] | 7 (41%) | 1.01 [0.77, 1.33] | 7 (53%) |
| Eczema by 1-3 years, by prescribed intervention timing | In first week of life | 0.95 [0.81, 1.12] | 6 (0%) | 0.99 [0.81, 1.20] | 5 (9%) |
|  | After first week of life | 1.57 [1.10, 2.23] | 1 (NA) | 0.89 [0.27, 2.95] | 2 (84%) |
|  | Total | 1.03 [0.81, 1.31 | 7 (41%) | 1.01 [0.77, 1.33] | 7 (53%) |
